# Identification of the *ABCC4*, *IER3*, and *CBFA2T2* candidate genes for resistance to paratuberculosis from sequence-based GWAS in Holstein and Normande dairy cattle

**DOI:** 10.1186/s12711-020-00535-9

**Published:** 2020-03-17

**Authors:** Marie-Pierre Sanchez, Raphaël Guatteo, Aurore Davergne, Judikael Saout, Cécile Grohs, Marie-Christine Deloche, Sébastien Taussat, Sébastien Fritz, Mekki Boussaha, Philippe Blanquefort, Arnaud Delafosse, Alain Joly, Laurent Schibler, Christine Fourichon, Didier Boichard

**Affiliations:** 1 Université Paris Saclay, INRAE, AgroParisTech, GABI, 78350 Jouy-en-Josas, France; 2 BioEpAR, INRAE, Oniris, CS, 40706 Nantes, France; 3 GDS Haute Normandie, 76000 Rouen, France; 4 Allice, 149 Rue de Bercy, 75012 Paris, France; 5 GDS Pays de Loire, 49800 Trélazé, France; 6 GDS Orne, 61000 Alençon, France; 7 GDS Bretagne, 56000 Vannes, France

## Abstract

**Background:**

Bovine paratuberculosis is a contagious disease, caused by *Mycobacterium avium* subsp. *paratuberculosis* (MAP), with adverse effects on animal welfare and serious economic consequences. Published results on host genetic resistance to MAP are inconsistent, mainly because of difficulties in characterizing the infection status of cows. The objectives of this study were to identify quantitative trait loci (QTL) for resistance to MAP in Holstein and Normande cows with an accurately defined status for MAP.

**Results:**

From MAP-infected herds, cows without clinical signs of disease were subjected to at least four repeated serum ELISA and fecal PCR tests over time to determine both infected and non-infected statuses. Clinical cases were confirmed using PCR. Only cows that had concordant results for all tests were included in further analyses. Positive and control cows were matched within herd according to their birth date to ensure a same level of exposure to MAP. Cows with accurate phenotypes, i.e. unaffected (control) or affected (clinical or non-clinical cases), were genotyped with the Illumina BovineSNP50 BeadChip. Genotypes were imputed to whole-genome sequences using the 1000 Bull Genomes reference population (run6). A genome-wide association study (GWAS) of MAP status of 1644 Holstein and 649 Normande cows, using either two (controls versus cases) or three classes of phenotype (controls, non-clinical and clinical cases), revealed three regions, on *Bos taurus* (BTA) chromosomes 12, 13, and 23, presenting significant effects in Holstein cows, while only one of those was identified in Normande cows (BTA23). The most significant effect was found on BTA13, in a short 8.5-kb region. Conditional analyses revealed that only one causal variant may be responsible for the effects observed on each chromosome with the *ABCC4* (BTA12), *CBFA2T2* (BTA13), and *IER3* (BTA23) genes as good functional candidates.

**Conclusions:**

A sequence-based GWAS on cows for which resistance to MAP was accurately defined, was able to identify candidate variants located in genes that were functionally related to resistance to MAP; these explained up to 28% of the genetic variance of the trait. These results are very encouraging for efforts towards implementation of a breeding strategy aimed at improving resistance to paratuberculosis in Holstein cows.

## Background

Paratuberculosis, also referred to as Johne’s disease (JD), is an infectious, contagious, and incurable disease caused by *Mycobacterium avium* subsp. *paratuberculosis* (MAP). Worldwide, no country is known to be free of MAP; it primarily affects domestic ruminants, and a high bovine herd prevalence (up to 50%) has been reported in Europe [1]. In cattle, while adult-to-calf transmission is possible in utero or via contaminated colostrum or milk, the main route of transmission occurs via the uptake of MAP-infected feces by calves. After a transient phase of shedding, calves undergo a latency phase that can last for several months or years. Subclinical symptoms of the disease include weight loss and reduced milk production together with a humoral immune response and fecal shedding; because of this, subclinical cases may continue to contaminate their environment for years. Clinical cases finally develop chronic diarrhea and severe emaciation, ending in death. Thus, JD has substantial adverse effects on animal welfare as well as a detrimental economic impact on the cattle sector [2]. There are no effective treatment protocols and vaccination (which provides only partial protection) is limited because it can induce a false positive reaction at the intradermal test used for tuberculosis detection [3]. For this reason, several countries have initiated programs for the control of JD with the objective of reducing MAP contamination, mainly by testing animals, culling seropositive animals and preventing the exposure of calves, which are the most susceptible animals in infected farms [4]. However, these programs have a high cost and a limited efficiency in clearing MAP mainly because of the disease’s long periods of latency (from infection to seropositive conversion or fecal shedding) and incubation (from infection to clinical symptoms) [5]. These periods also vary greatly among individuals, as does the amount of bacteria shed by infected cattle, which suggests the existence of individual variability in resistance to MAP.

As recently reviewed by Brito et al. [6], various studies have shown the role of host genetics in resistance to MAP and have estimated heritability values for this trait between 0.03 and 0.27. In addition, numerous genome-wide association studies (GWAS) have been conducted using genotyping of single-nucleotide polymorphisms (SNPs) at low [7], medium [8–14], or high [6, 15, 16] densities as well as whole-genome sequences [17, 18] imputed from data of the 1000 Bull Genomes project [19]. These GWAS analyses have led to the identification of quantitative trait loci (QTL) associated with resistance/susceptibility to MAP on all *Bos taurus* (BTA) autosomes, with the most frequently identified QTL located on BTA1, 6, 7, and 23.

Unfortunately, the results of genetic analyses (heritability estimates and QTL identification) differ quite a bit among published studies. These inconsistencies may arise from multiple factors such as differences in the population analyzed, disease prevalence in the population, the statistical model applied, and the definition used for the MAP-resistant phenotype. The studies mentioned above generally defined phenotypes based on a single measurement of serum ELISA, milk ELISA, MAP culture in feces or tissue, or PCR on feces, which may result in an incorrect diagnosis due to (i) the lack of sensitivity of these tests [20], (ii) the lack of concordance between ELISA and fecal culture tests [21], (iii) the potential misidentification of unexposed individuals as resistant, and (iv) variability in longitudinal serological and fecal shedding patterns among animals [5].

To avoid these drawbacks, the present work was designed as a case–control study based on confirmed phenotypes. Only confirmed positive or negative individuals were included, based on the criteria previously defined from a longitudinal study of MAP fecal shedding and serological patterns in dairy cattle [5]. To avoid a putative bias of non-exposure, the negative animals were born in the same herd and in the same period as the positive animals. In total, 1644 Holstein and 649 Normande cows with relevant and accurate contrasted phenotypic profiles (control and non-clinical/clinical cases) were genotyped with a medium-density SNP chip (50 K SNP); whole-genome sequences (WGS) were subsequently imputed using the 1000 Bull Genomes reference population (run6) [19]. Here, we present the results obtained from GWAS analyses conducted in both Holstein and Normande cows on the imputed WGS datasets.

## Methods

### Animals and phenotypes

**Fig. 1:**
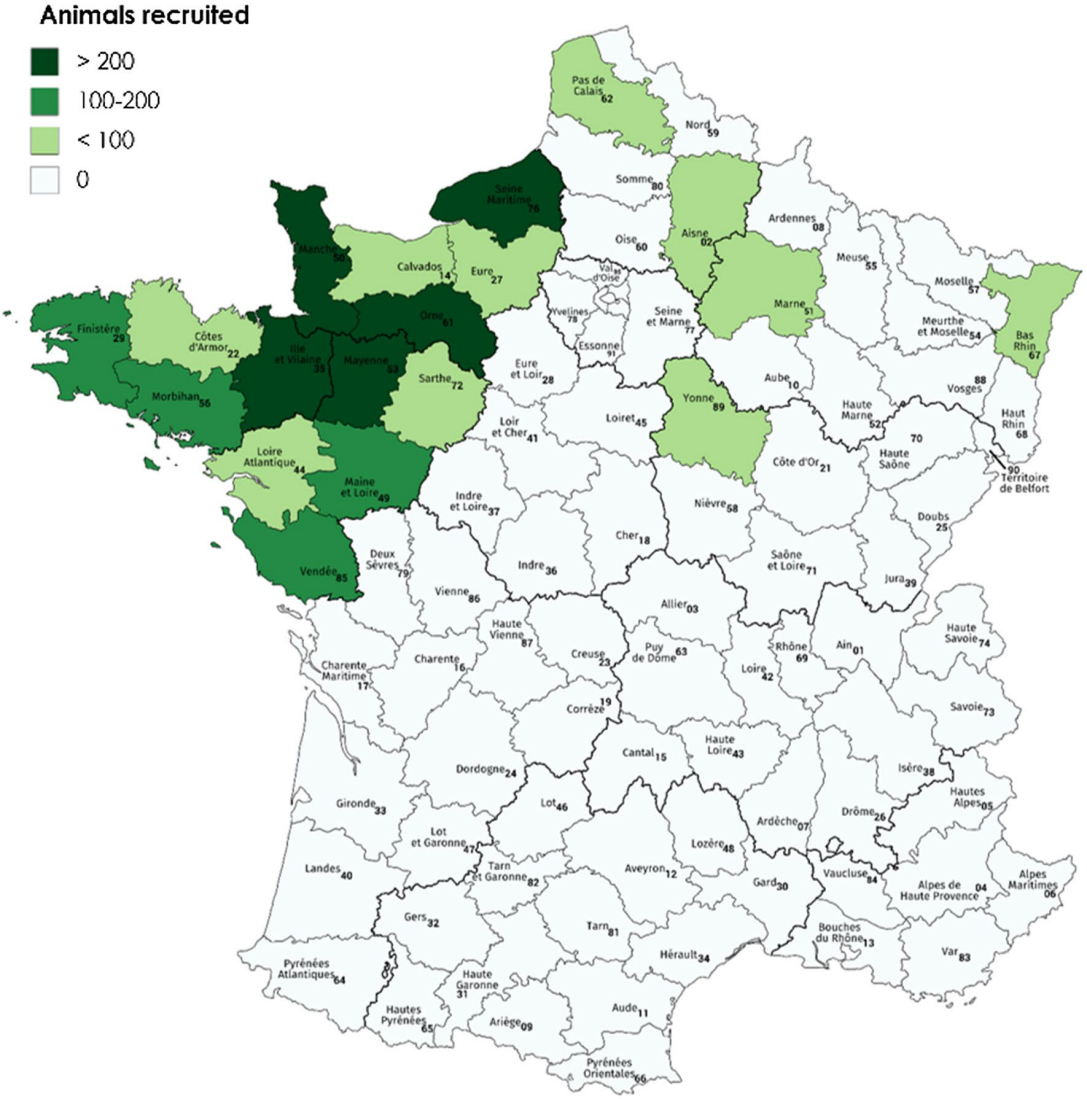
Locations of herds enrolled in control plans and number of cows recruited

Animals of certified parentage were recruited from 2034 French herds enrolled in paratuberculosis control plans. From these herds, mainly located in the northwestern region of France (Fig. 1), cows were recruited based on their serological status. Serum samples were analyzed by ELISA (Idexx, Montpellier, France, or Idvet, Montpellier, France). Based on a prior study [5], tests were considered positive if the S/P value (sample optical density over positive control optical density, as a percentage) was 90% or higher, and negative if S/P was lower than 45% (Idexx) or 60% (Idvet). For each cow, at least two ELISA tests were carried out, with at least 8 months between the tests. Strict conditions had to be met for negative cows to be included as controls. They had to be at least 48 months old, to exclude animals still in the latency period. In addition, only cows born in the same herd and the same period (± 1.5 months) as affected cows were retained, in order to maximize the potential for exposure to MAP. All animals with conflicting results were removed from the analysis. All cows that presented concordant serological statuses, either positive or negative, were then subjected to additional standardized ELISA and PCR analyses in a single laboratory (Laboratoire Départemental du Bas-Rhin, LD67, Strasbourg, France) with the same test kits throughout the experiment (Idexx for serology; Adiagene (Saint Brieuc, France) for PCR MAP quantification in the feces); an animal was only retained if its subclinical status or control phenotype was confirmed. Cows were considered to be shedders if the qPCR threshold cycle (C_t_) was 35 or less, and non-shedders if it was 40 or more. All samples with intermediate results (35 < C_t_ < 40) were considered to be of uncertain status and were excluded from the study. Regarding the Elisa test, the threshold of 45 was used. Both tests had to provide concordant results. Based on these requirements, 1465 control cows were confirmed and 658 were eliminated; and 1138 subclinical cases were confirmed and 580 were eliminated. In addition, clinical cases (245 Holstein and 74 Normande) were identified by clinical diagnosis and confirmed by the presence of MAP in feces. Finally, following this rigorous protocol, the statuses of 2453 cows (1759 Holstein and 694 Normande), for which well conserved blood samples were available for DNA extraction, were confirmed as positive non-clinical or clinical cases or negative controls. These phenotypes were coded in subsequent analyses in one of two ways: (i) 0/1/2 for controls, non-clinical cases, and clinical cases, respectively, and (ii) 0/1 for controls and cases (non-clinical and clinical), respectively.

### Genotyping and imputation to whole-genome sequences

Animals with confirmed phenotypes were genotyped using the BovineSNP50 (50 K, Illumina Inc., San Diego) Beadchip. After quality control, 43,801 autosomal SNPs were retained. The filters used were those applied in the French national evaluation system [22]: individual call rate higher than 95%, SNP call rate higher than 90%, minor allele frequency (MAF) higher than 1% in at least one major French dairy cattle breed, and genotype frequencies in Hardy–Weinberg equilibrium with *P *> 10^−4^. Animals that were incompatible with their parents (when all genotypes were available) were excluded. In the end, 2293 cows (1644 Holstein and 649 Normande) were kept for the genetic study (Table 1).

**Table 1:** Number of Holstein and Normande cows with confirmed phenotypes

Phenotype	Criteria	Number of Holstein	Number of Normande	Total number
Control (0)	≥ 2 ELISA S/P < 45%*	838	233	1071
	1 Idexx ELISA S/P < 45%			
	1 qPCR Ct ≥ 40			
Non-clinical case (1)	≥ 2 ELISA S/P ≥ 90%	577	347	924
	1 Idexx ELISA S/P > 45%			
	1 qPCR Ct ≤ 35			
Clinical case (2)	Clinical signs and presence of MAP in feces	229	69	298
Total		1644	649	2293

*2 ELISA performed at the regional level, thresholds were 45% with Idexx and 60% with Idvet test

Then, the 50 K SNP genotypes were imputed to whole-genome sequences (WGS). A two-step approach was applied in order to improve the accuracy of imputed genotypes of the WGS variants [23]: from 50 to 777 K high-density (HD) SNPs using FImpute software [24], and then from imputed HD SNPs to WGS, using the Minimac software [25]. In spite of a longer computing time, Minimac was preferred to FImpute for the imputation of WGS because it infers allele dosages in addition to the best-guess genotypes. Compared to best-guess genotypes, allele dosages are expected to be more correlated to the true genotypes [26] and to lead to better targeting of causative mutations in GWAS analyses [27]. Imputations from 50 K to the HD SNP level were performed using within-breed reference sets of 776 Holstein and 546 Normande bulls that were genotyped with the Illumina BovineHD BeadChip (Illumina Inc., San Diego, CA) [28]. WGS variants were imputed from HD SNP genotypes using WGS of 2333 *Bos taurus* animals, from the 6th run of the 1000 Bull Genomes Project [26, 29]. These animals represent 51 cattle breeds and include 544 Holstein and 44 Normande individuals. In the Normande breed, the number of animals with whole-genome sequences was rather limited but we identified most of them as influential ancestor bulls with a high cumulated contribution to the breed (74%). We applied the protocol described in [30] and produced 23,781,173 and 23,610,986 autosomal variants for the Holstein and Normande cows, respectively. The precision of imputation from HD SNP to sequence level was assessed using the coefficient of determination (R^2^) calculated with the Minimac software [25]. In order to remove variants with low imputation accuracies, only variants with an R^2^ value higher than 30% and a MAF higher than 1% were retained for further association analyses, i.e. 7,914,731 and 7,673,760 variants for Holstein and Normande, respectively, with a mean R^2^ close to 81% (R^2^ classes between 30 and 80% included a limited number of variants).

### Whole-genome sequence association analyses

We performed single-trait association analyses between all variants and the MAP resistance/susceptibility phenotypes (0/1/2 and 0/1). All association analyses were performed using the *mlma* option of the GCTA software (version 1.24), which applies a mixed linear model that includes the variant to be tested [31]. The categorical nature of the phenotype (2 or 3 classes) was not accounted for in this model assuming a normal distribution.

1 $${\mathbf{y}} = {\mathbf{1}}{\upmu} { + }{\mathbf{x}}{\text{b}} + {\mathbf{u}} + {\mathbf{e}} ,$$

where ${\text{y}}$ is the vector of individual phenotypes; $\upmu$ is the overall mean; ${\text{b}}$ is the additive fixed effect of the variant to be tested for association; ${\mathbf{x}}$ is the vector of predicted allele dosages, varying between 0 and 2; ${\mathbf{u}}\sim N\left( {{\mathbf{0}},{\mathbf{G}}\upsigma _{{\text{u}}}^{2} } \right)$ is the vector of random polygenic effects, with ${\mathbf{G}}$ the genomic relationship matrix (GRM) that is calculated by using the HD SNP genotypes [32], and $\upsigma_{\text{u}}^{2}$ the polygenic variance that is estimated based on the null model ${\mathbf{y}} = {\mathbf{1}}\upmu + {\mathbf{u}} + {\mathbf{e}}$ and then fixed while testing for the association between each variant and the trait of interest; and ${\mathbf{e}}\sim N\left( {{\mathbf{0}},{\mathbf{I}}\upsigma _{{\text{e}}}^{2} } \right)$ is the vector of random residual effects, with ${\mathbf{I}}$ the identity matrix and $\upsigma_{\text{e}}^{2}$ the residual variance. No other fixed effect was included in the model. Indeed, because of the experimental design, it was not possible to test the usual factors of variation such as herd effect (only a few selected records retained per herd) or effects of age or season.

In order to account for multiple testing, we estimated the number of independent chromosome segments $N_{S} = 9600$ by applying the formula of Goddard [33] $N_{S} = 4 N_{e} L$, with $N_{e} = 80$, the effective population size and $L = 30$, the length of the genome in Morgans. We assumed that five SNPs were needed to saturate and summarize each segment and we applied the Bonferroni correction to the thresholds by considering 50,000 independent tests. Therefore, the 5% genome-wide threshold of significance corresponded to a nominal *P* value of 10^−6^ (−log_10_(*P*) = 6). After identification of the lead variant in a given region, variants with significant effects that were located less than 500 kbp apart were grouped in the same QTL region. Then, the boundaries of QTL regions were determined by considering the positions of variants that were included in the upper third of the peak. The process was repeated with the next top variants. When two consecutive QTL regions had overlapping confidence intervals, they were grouped in a unique QTL region. Variants that were located within QTL regions were annotated using Variant Effect Predictor (VEP) [34] based on the UMD3.1 bovine reference genome assembly.

### Conditional association analyses

In order to determine if the presence of multiple significant variants in a genomic region was a reflection of distinct causal mutations or due to linkage disequilibrium (LD) with a single causal mutation, conditional analyses were carried out in the most significant QTL regions using the *cojo* option of GCTA [35]. Conditional association analyses were performed by including in the model the most significant variant as a fixed effect and by testing all variants that were not in strong LD with the conditional variant (r^2^ < 0.9).

## Results

### GWAS results

Genetic and residual variances were estimated using the genomic relationship matrices calculated from HD genotypes. Heritability estimates for phenotypes 0/1 or 0/1/2 were similar and high for both Normande and Holstein cows; they ranged from 0.51 to 0.57 depending on the phenotype and on the breed (Table 2).

**Table 2:** Variance and heritability values estimated from the genomic relationship matrix calculated using HD genotypes

Breed	Phenotype	Genetic variance	Residual variance	h^2^ (SE)^a^
Normande	0/1	0.12	0.12	0.51 (0.15)
	0/1/2	0.21	0.20	0.52 (0.15)
Holstein	0/1	0.14	0.10	0.57 (0.09)
	0/1/2	0.29	0.21	0.57 (0.09)

^a^Note that these heritability estimates are likely to be strongly overestimated, due to the selection of extreme phenotypes (see Discussion)

**Fig. 2:**
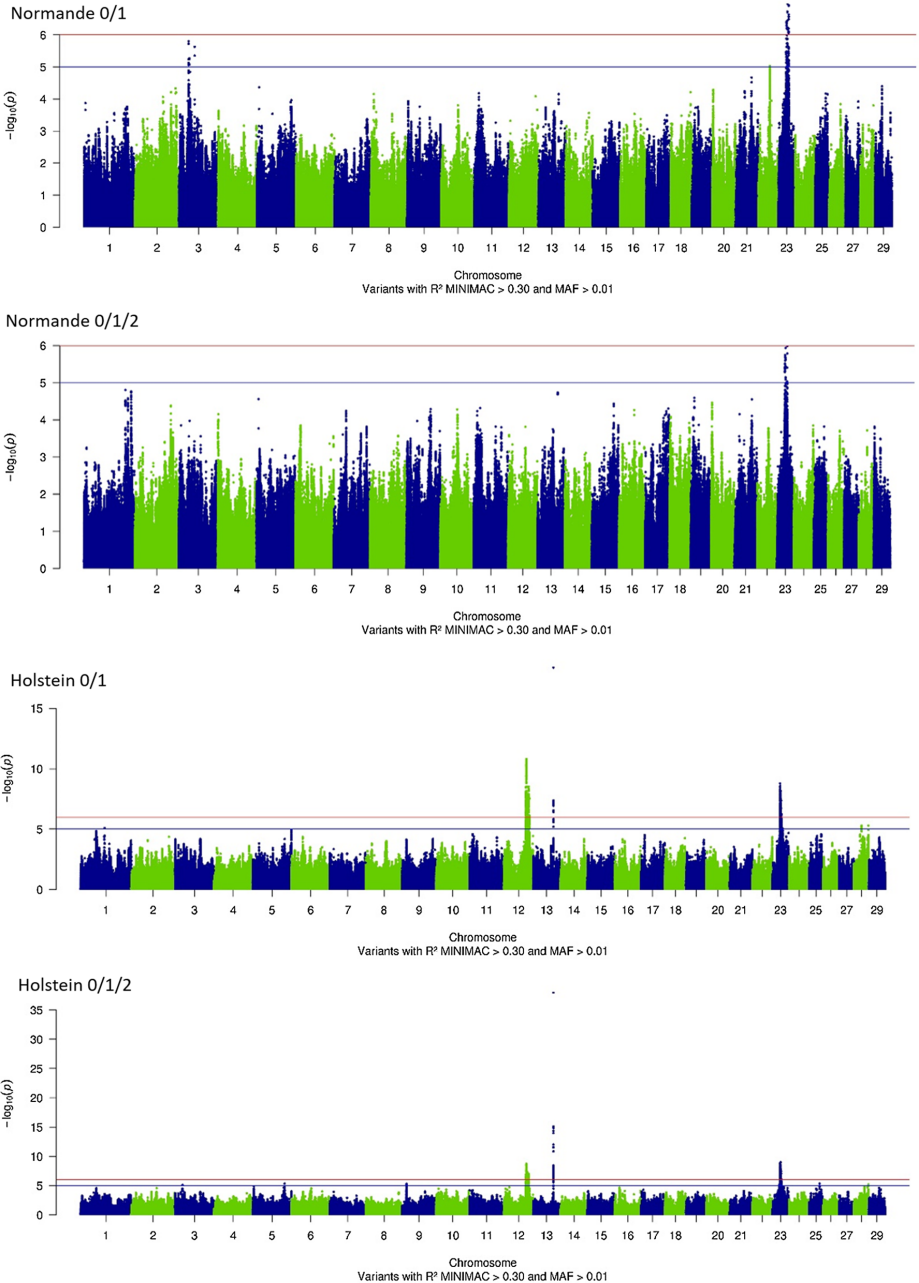
−log_10_(*P*) values plotted against the position of variants on *Bos taurus* (BTA) autosomes for the 0/1 and 0/1/2 phenotypes of Normande and Holstein cows

Then, we tested the significance of effects for more than 7 million variants in Normande and Holstein cows for both the 0/1 and 0/1/2 phenotypes. In total, 2446 variants (SNPs or indels) presented significant effects (−log_10_(*P*) ≥ 6) in at least one analysis (Fig. 2). In Normande cows, we identified 41 variants with significant effects for the 0/1 phenotype, but no variant reached the level of significance for the 0/1/2 phenotype. In Holstein, 1566 and 1719 variants had significant effects for phenotypes 0/1 and 0/1/2, respectively. Variants with significant effects were located on BTA23 in Normande cows and on BTA12, 13, and 23 in Holstein cows (Fig. 2). In the Holstein breed, variants that exhibited the most significant effects were located on BTA13, with a −log_10_(*P*) value up to 38.1 for the 0/1/2 phenotype.

**Fig. 3:**
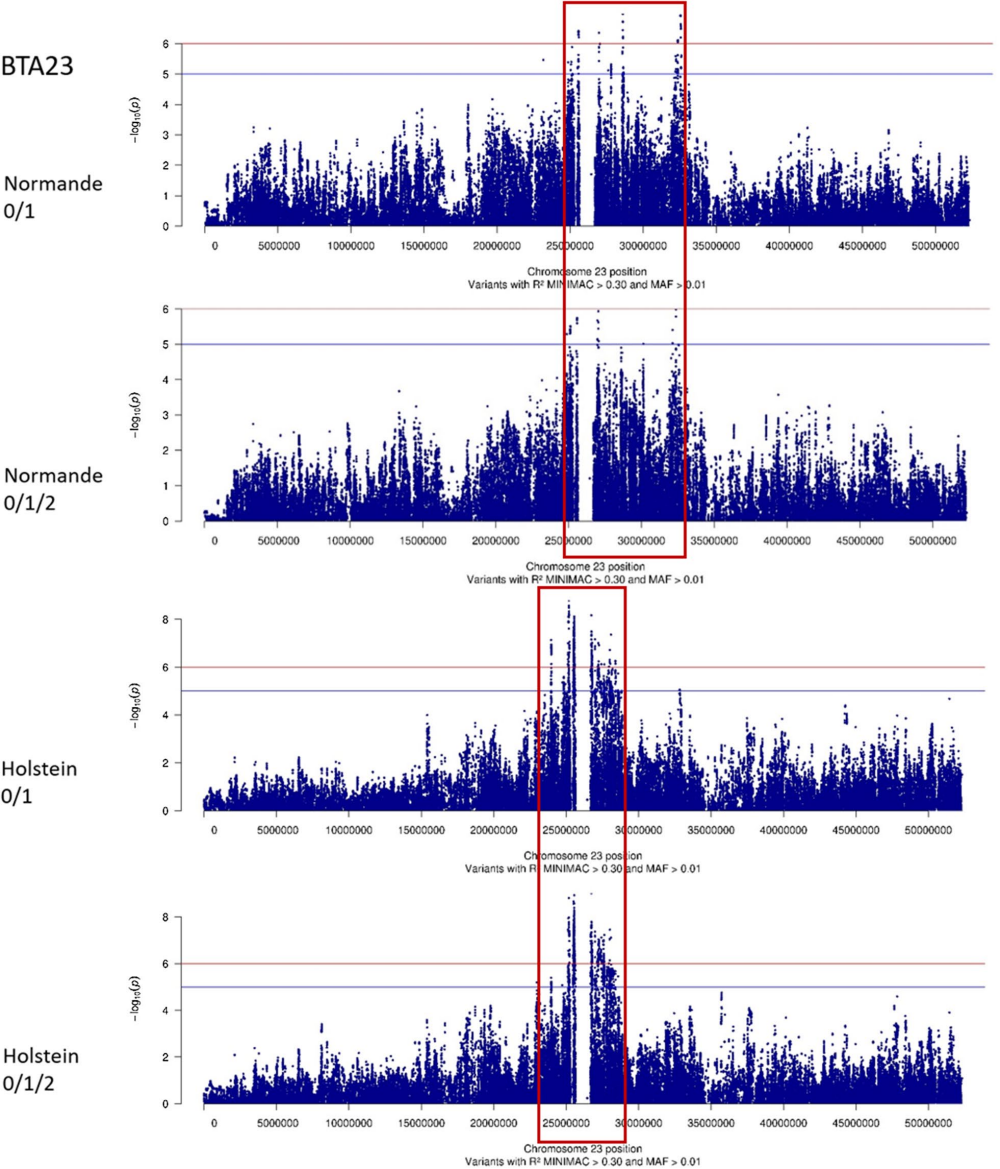
-log_10_(*P*) values plotted against the position of variants on *Bos taurus* (BTA) autosome 23 for the 0/1 and 0/1/2 phenotypes of Normande and Holstein cows

All variants with significant effects were grouped into four, eleven, and nine QTL for the Normande 0/1, Holstein 0/1, and Holstein 0/1/2 analyses, respectively (Table 3). The four QTL found in Normande cows for the 0/1 phenotype were located between 24.8 and 32.6 Mbp on BTA23 (Fig. 3). As shown in Fig. 3, the effects of this region were also very close to significance for the 0/1/2 phenotype in the same breed (−log_10_(*P*) = 5.99). In neighboring regions on BTA23, we also identified four QTL for the 0/1 phenotype (23.5–28.8 Mbp) and three QTL for the 0/1/2 phenotype (25.1–28.4 Mbp) in Holstein cows.

**Table 3:** Description of the QTL identified in GWAS analyses for the 0/1 or 0/1/2 phenotypes of Normande and Holstein cows

Breed	Phenotype	QTL	BTA	Confidence interval				TOP variant (resistance allele)							
				Start (bp*)	End (bp*)	Number of significant variants	Number of genes	−log_10_(*P*)	ID	bp*	Freq.	Effect	SE	Gene	Functional annotation
Normande	0/1	1	23	24,804,034	25,602,017	7	3	6.4	rs134517431	25,575,732	0.43	− 0.17	0.03	*BOLA*-*DRB2*	Intron
Normande	0/1	2	23	26,952,214	27,814,232	1	26	6.4	rs110031509	26,977,608	0.37	− 0.15	0.03	*NOTCH4*	Synonymous
Normande	0/1	3	23	28,576,812	28,671,569	7	2	7.0	rs208517124	28,605,243	0.31	− 0.17	0.03	*TRIM15*	Intron
Normande	0/1	4	23	32,128,351	32,606,331	25	0	6.9	rs108942026	32,551,869	0.27	− 0.17	0.03	–	Intergenic
Holstein	0/1	1	12	68,908,056	69,745,891	268	2	9.4	rs378812903	69,314,330	0.81	− 0.16	0.03	–	Intergenic
Holstein	0/1	2	12	69,774,746	71,240,801	281	1	10.8	rs43161232	70,723,087	0.88	− 0.19	0.03	–	Intergenic
Holstein	0/1	3	12	72,272,367	72,416,273	144	0	8.5	rs43047348	72,351,583	0.77	− 0.15	0.03	–	Intergenic
Holstein	0/1	4	12	75,283,615	75,380,545	6	0	7.7	rs43104474	75,379,770	0.90	− 0.18	0.03	–	Intergenic
Holstein	0/1	5	12	76,760,851	77,323,511	215	1	8.5	rs378568583	77,285,189	0.76	− 0.14	0.02	–	Intergenic
Holstein	0/1	6	12	79,096,880	80,067,268	6	1	7.5	rs379488452	80,064,150	0.84	− 0.14	0.03	*UBAC2*	Intron
Holstein	0/1	7	13	63,497,960	63,506,532	20	0	18.5	rs109570209	63,502,566	0.91	− 0.34	0.04	–	Intergenic
Holstein	0/1	8	23	23,529,491	23,976,432	39	1	7.1	rs210372926	23,956,987	0.34	− 0.11	0.02	*PKHD1*	Intron
Holstein	0/1	9	23	25,115,933	25,582,101	360	3	8.8	rs209183236	25,181,661	0.66	− 0.12	0.02	*ELOVL5*	Intron
Holstein	0/1	10	23	26,665,220	27,629,951	114	28	8.2	rs470365293	26,733,104	0.69	− 0.17	0.03	*ENSBTAG00000023541*	Intron
Holstein	0/1	11	23	27,630,248	28,817,610	13	5	7.4	rs109539043	28,085,410	0.81	− 0.13	0.02	*IER3*	Upstream
Holstein	0/1/2	1	12	67,954,912	69,097,427	31	0	6.9	rs110051821	68,949,675	0.67	− 0.21	0.04	–	Intergenic
Holstein	0/1/2	2	12	69,162,598	70,723,087	267	3	8.7	rs41667085	70,127,519	0.91	− 0.28	0.05	*ABCC4*	Intron
Holstein	0/1/2	3	12	71,239,317	71,240,801	3	0	6.7	rs382007737	71,240,801	0.74	− 0.25	0.05	–	Intergenic
Holstein	0/1/2	4	12	72,272,367	72,415,271	125	0	7.4	rs463644618	72,356,381	0.95	− 0.56	0.10	–	Intergenic
Holstein	0/1/2	5	12	76,760,851	77,323,511	65	1	7.0	rs383583316	77,253,589	0.76	− 0.17	0.03	–	Intergenic
Holstein	0/1/2	6	13	63,497,960	63,506,532	20	0	38.1	rs109570209	63,502,566	0.91	− 0.71	0.05	–	Intergenic
Holstein	0/1/2	7	23	25,119,808	25,602,067	456	3	8.9	rs210655104	25,554,248	0.83	− 0.24	0.04	–	Intergenic
Holstein	0/1/2	8	23	26,680,418	27,629,951	345	28	9.0	rs470365293	26,733,104	0.69	− 0.26	0.04	*ENSBTAG00000023541*	Intron
Holstein	0/1/2	9	23	27,759,542	28,398,451	9	3	7.4	rs209284762	28,012,299	0.84	− 0.21	0.04	–	Intergenic

* Positions of variants on UMD3.1

**Fig. 4:**
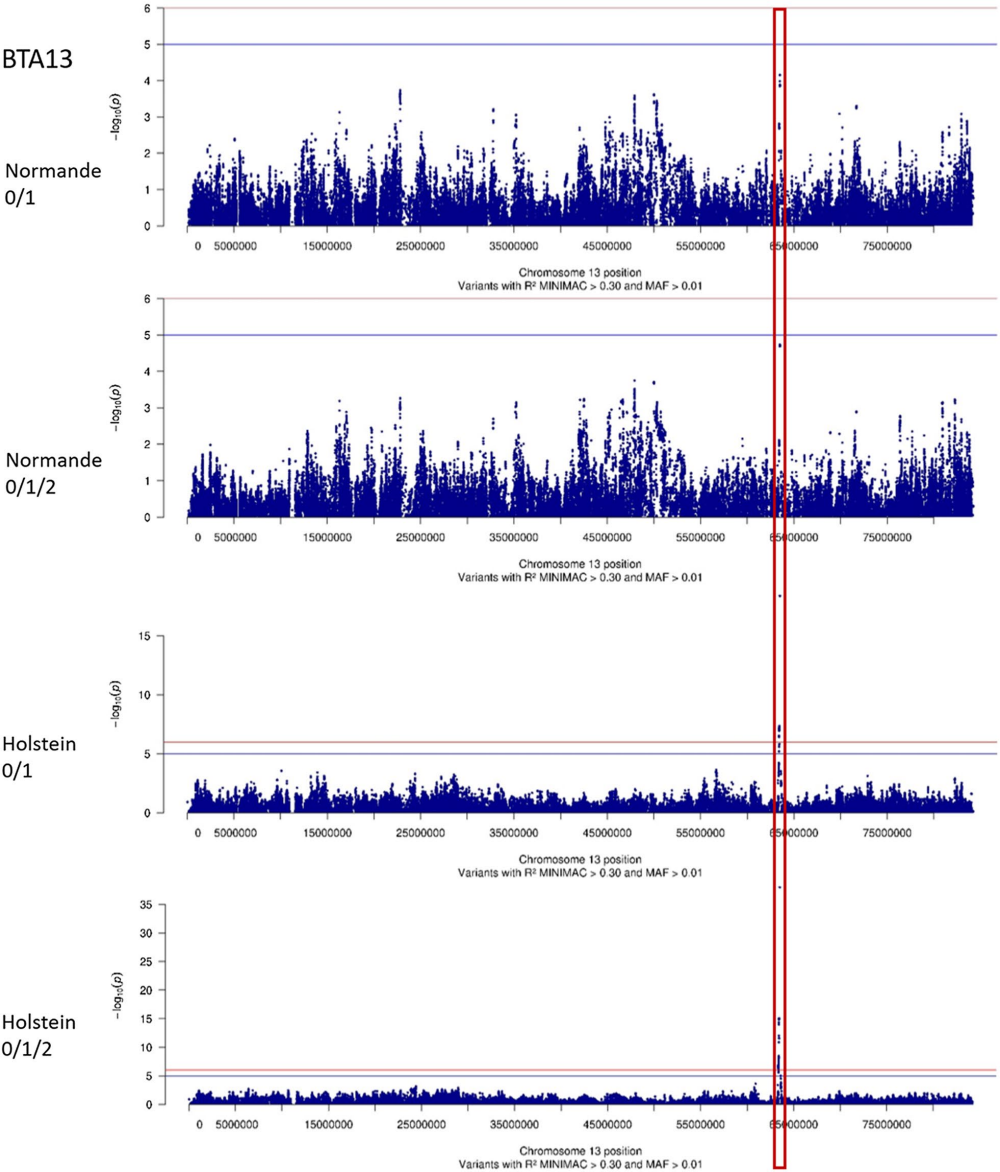
−log_10_(*P*) values plotted against the position of variants on *Bos taurus* (BTA) autosome 13 for the 0/1 and 0/1/2 phenotypes of Normande and Holstein cows

**Fig. 5:**
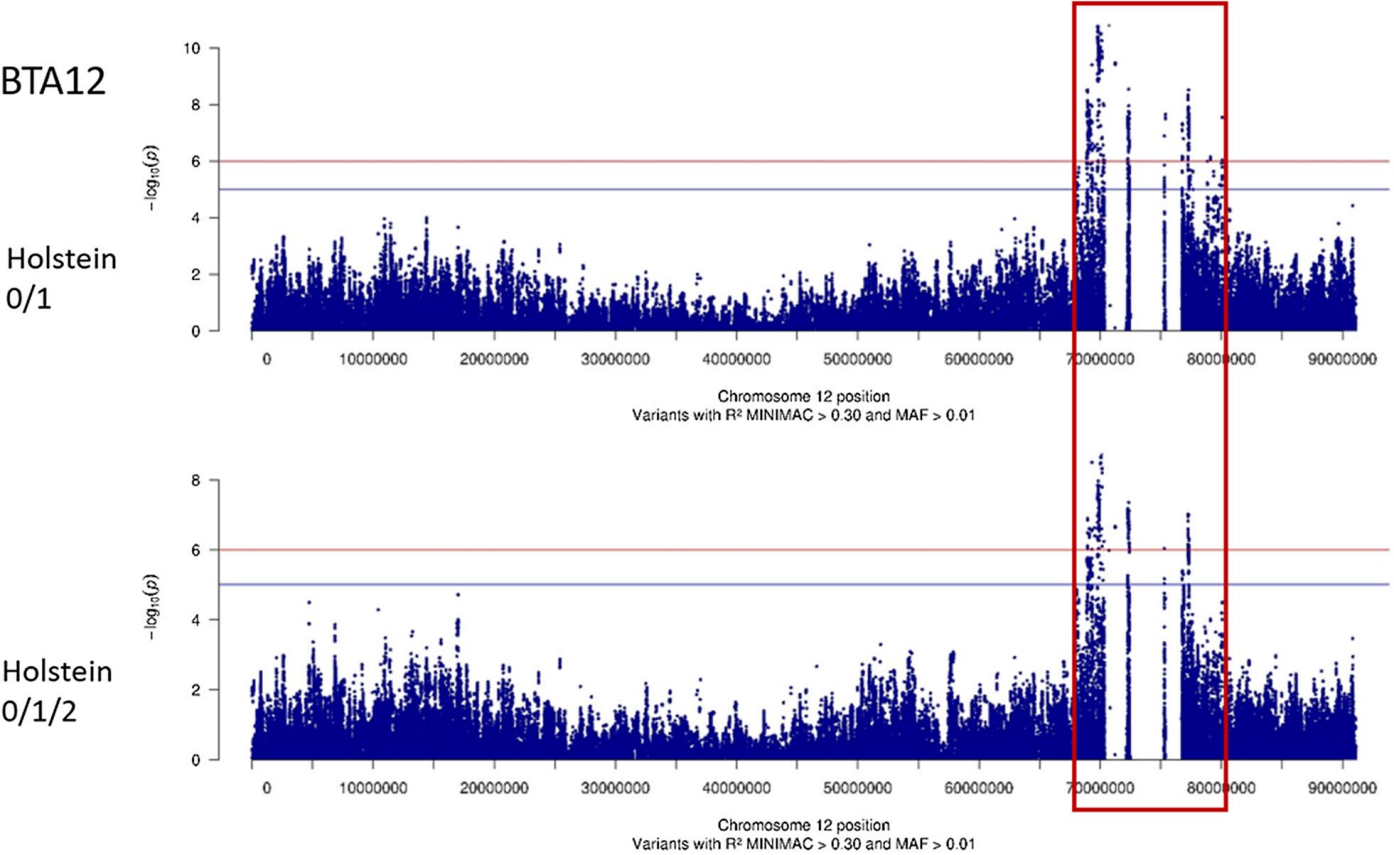
−log_10_(*P*) values plotted against the position of variants on *Bos taurus* (BTA) autosome 12 for the 0/1 and 0/1/2 phenotypes of Holstein cows

However, in Holsteins, the most significant effects were found on BTA13 (Fig. 4) and to a lesser extent on BTA12 (Fig. 5). The QTL detected on BTA13 was located in a very narrow 8-kpb region, and the variant showing the most significant effects (rs109570209) was located at 63,502,566 bp in both the 0/1 and 0/1/2 analyses. Moreover, although no significant QTL was observed in this region in Normande cows, the analysis revealed a narrow peak that was located exactly in the same region, and the variants with the highest −log_10_(*P*) values were located at 63,502,649 and 63,502,566 bp (Fig. 4). Finally, in Holstein cows, the remaining QTL were located on BTA12. For the 0/1 phenotype, six distinct QTL were located between 68.9 and 80.1 Mbp, while for the 0/1/2 phenotype five QTL were detected between 67.9 and 77.3 Mbp (Fig. 5).

**Fig. 6:**
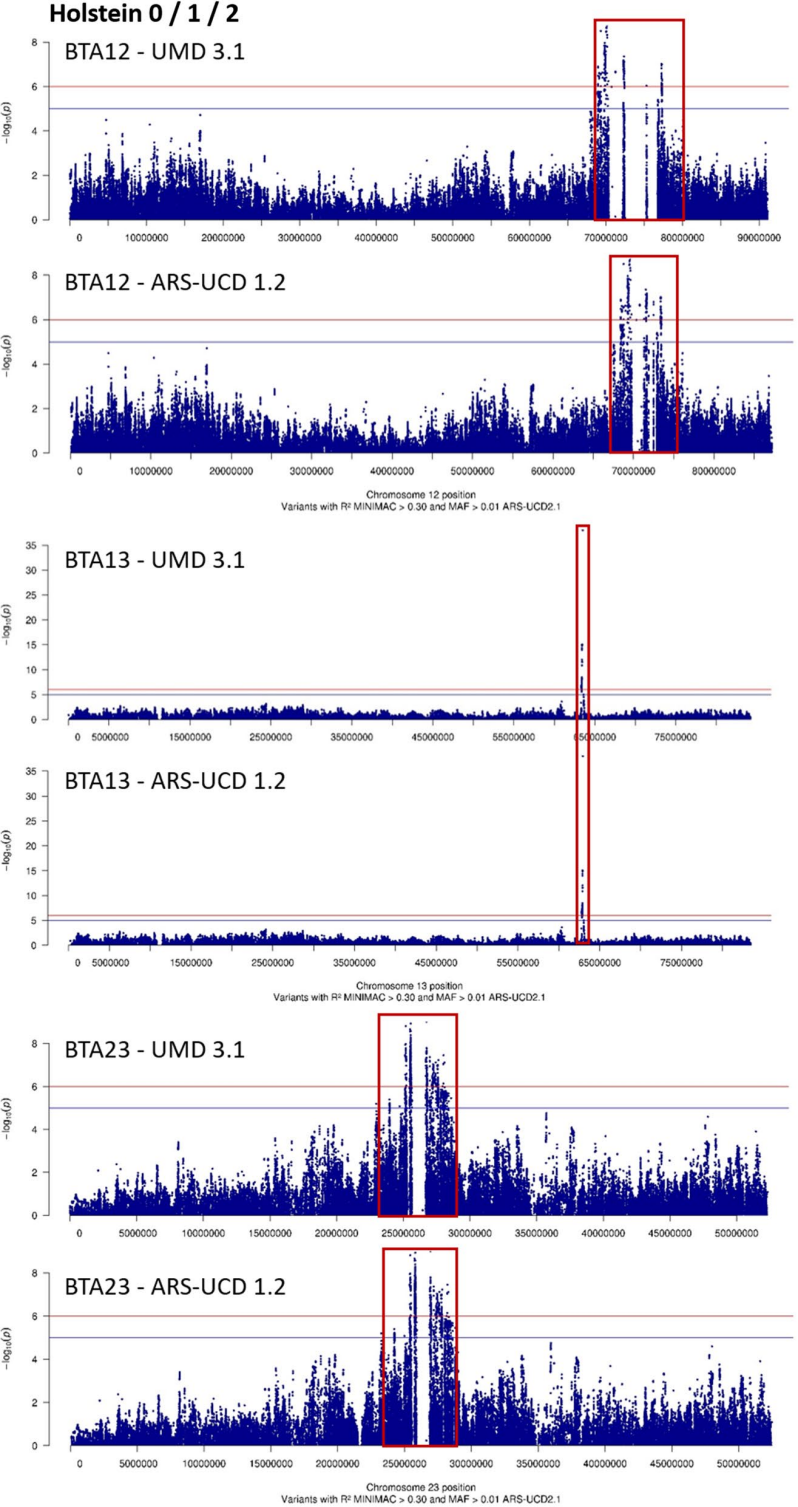
−log_10_(*P*) values plotted against the position of variants on UMD3.1 and ARS-UCD1.2 assemblies of *Bos taurus* (BTA) autosomes 12, 13, and 23 for the 0/1/2 phenotype of Holstein cows

It should be noted that these results were based on the UMD3.1 bovine reference genome assembly. In this assembly, the QTL regions on BTA12 and 23 are of limited quality and the corresponding number of QTL may be overestimated. Thus, we realigned these regions on the most-recent ARS-UCD1.2 assembly, with the results presented in Fig. 6. For the QTL on BTA13 and 23, we found few changes, i.e. the most-recent assembly was very similar to the UMD3.1 assembly for the variants located in these regions. In contrast, on BTA12, the analysis with the most recent assembly detected a smaller number of QTL (e.g., three for the 0/1/2 phenotype in Holstein versus five with the UMD3.1 assembly) located within a shorter interval (5 Mbp with ARS-UCD1.2 versus 9.4 Mbp with the UMD3.1 assembly).

### Functional annotations

Functional annotations revealed that 74% of the 1868 distinct variants located within the confidence intervals of the QTL were intergenic (65% for 0/1 in Normande, 81% for 0/1 in Holstein, and 70% for 0/1/2 in Holstein) (Table 4). Therefore, the remaining variants were located in genes, mainly in introns (16%) and upstream regions (7%), and more rarely in downstream regions (1.4%), exons (0.5% missense and 0.5% synonymous), 3′ UTR regions (0.3%), and 5′ UTR regions (0.1%).

**Table 4:** Functional annotations of variants with significant effects (−log_10_(*P*) ≥ 6) located within confidence intervals of the QTL

Functional annotation	Normande 0/1	Holstein 0/1	Holstein 0/1/2	Total distinct
Intergenic	26	1189	930	1384
Intronic	11	232	218	303
Upstream	2	34	126	130
Downstream	0	8	24	26
3′ UTR	0	2	5	5
5′ UTR	0	0	2	2
Synonymous	1	1	8	9
Missense	0	1	9	9
Total	40	1467	1322	1868

Depending on the breed and the phenotype analyzed, the QTL linked with MAP resistance/susceptibility had confidence intervals that ranged in size from 1.5 kbp to 1.6 Mbp, and contained between 3 and 456 variants with significant effects. A minority of the QTL detected were located entirely in intergenic regions (one QTL in the Normande 0/1 analysis, on BTA23; three QTL in the Holstein 0/1 analysis, two on BTA12 and one on BTA13; and four QTL in the Holstein 0/1/2 analysis, three on BTA12 and one on BTA13), while all other QTL contained variants located in 1 to 28 distinct genes.

**Fig. 7:**
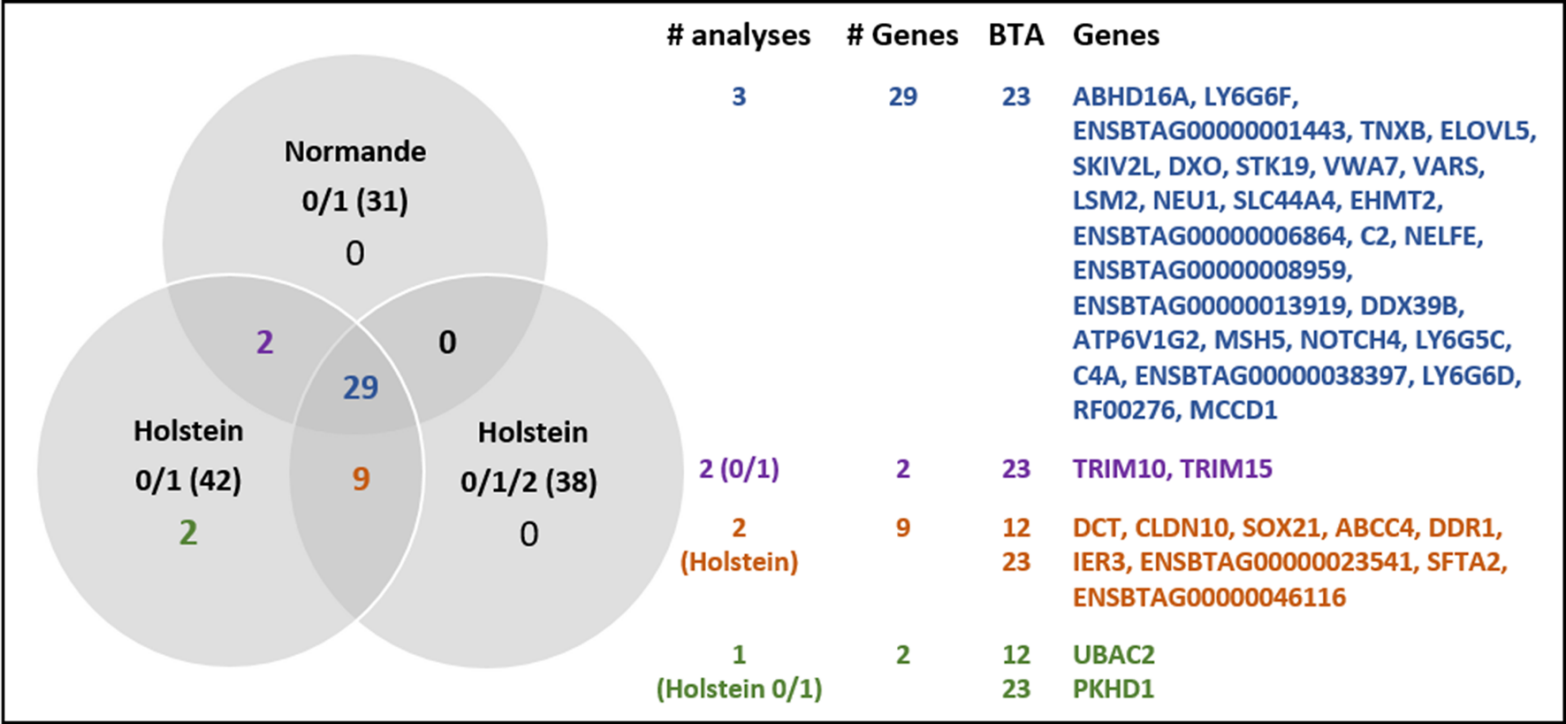
Number of overlapping candidate genes located in confidence intervals of QTL among Normande 0/1, Holstein 0/1, and Holstein 0/1/2 analyses and lists of corresponding genes

All the QTL found on BTA23 in the three analyses were located in the major histocompatibility complex (MHC) region, which is known to be particularly gene-rich. In each of the three analyses, the QTL that contained the largest number of genes were located around 27 Mbp. Here, within an interval of 1 Mbp, 26 and 28 genes were detected in Normande and Holstein cows, respectively. Although a large number of genes was located on BTA23, we identified a limited number of positional candidate genes within the confidence intervals of all of the QTL that we detected: 31, 42, and 38 in the Normande 0/1, Holstein 0/1, and Holstein 0/1/2 analyses, respectively (Fig. 7). The majority of these genes (29) were located on BTA23 and found in all three analyses. Two other genes, also located on BTA23, were shared in the 0/1 analyses of both breeds, while nine genes (4 on BTA12 and 5 on BTA23) appeared to be specific to the Holstein breed only (0/1 and 0/1/2 phenotypes), and two genes were identified in only one analysis (Holstein 0/1).

**Fig. 8:**
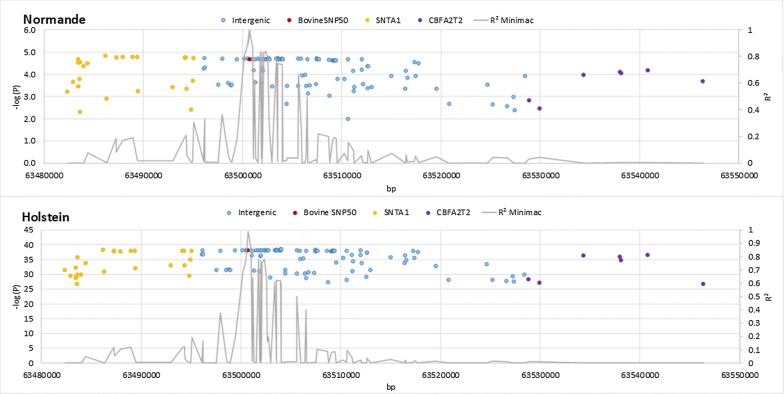
−log_10_(*P*) (dots) and R^2^ (line) values, calculated by Minimac, plotted against the position of variants in the [63,480,000-63,550,000] interval on *Bos taurus* (BTA) autosome 13 for the 0/1/2 phenotype of Normande and Holstein cows. Intergenic variants in blue, Bovine SNP50 variant in red, *SNTA1* genic variants in yellow, and *CBFA2T2* genic variants in purple

Therefore, we were able to identify positional candidate genes in all QTL regions except the QTL located on BTA13, i.e. the region that presented the most significant effects in Holstein cows. The confidence interval of this QTL, which was very narrow, contained only intergenic variants. However, two genes are located in the upstream region (*SNTA1* at 63,408,786–63,490,256 bp) and in the downstream region (*CBFA2T2* at 63,632,327–63,670,697 bp) of this QTL. The results discussed so far were obtained following the removal of variants with poor imputation accuracy or low MAF. When, instead, we considered all the variants of this region, including those imputed with poor accuracy (R^2^ < 0.3) and/or with a MAF < 0.01, we obtained another picture of the GWAS results. Figure 8 presents these results for the 0/1/2 phenotype in both Normande and Holstein cows. In this region, variants with the highest imputation accuracies were located in the vicinity of the BovineSNP50 variant (rs110002750 at 63,500,701 bp) in the intergenic region between the *SNTA1* and *CBFA2T2* genes. The closest variants that were located in genes were imputed with a very low accuracy and were therefore excluded from the GWAS analysis. However, their significance levels were similar to those found for the intergenic variants with the most significant effects. Therefore, it is likely that the confidence interval of this QTL also includes these variants with low imputation R^2^, and is larger than initially indicated (~ 70 kb vs 1.5 kb), meaning that this QTL probably also comprises *SNTA1* and *CBFA2T2*.

### Conditional GWAS

In order to distinguish multiple true causal mutations in a QTL region from those caused by LD, the variants with the most significant effects in each region, namely the top variants in Table 3 (11, 1, and 10 distinct variants for BTA12, 13, and 23, respectively), were tested in conditional analyses. Each of the top variants was individually included in the mixed model (1) as a fixed effect in addition to the variant to be tested in analyses of Holstein cows for both the 0/1 and 0/1/2 phenotypes (see Additional file 1: Figure S1).

Of the 11 variants added in the model for BTA12, only one removed the effects of all other variants tested on this chromosome, and only for the 0/1/2 phenotype. This variant was located at 70,127,519 bp (rs41667085) in an intronic region of the *ABCC4* gene, which encodes the multidrug resistance-associated protein 4, and was the variant on BTA12 that presented the most significant effect on the 0/1/2 phenotype (−log_10_(*P*) = 8.7). Interestingly, for the 0/1 phenotype, it ranked 62nd in the peak (−log_10_(*P*) = 10.3); instead, the variant with the most significant effect on this phenotype was located at 70,723,087 bp (−log_10_(*P*) = 10.8). For the 0/1 phenotype, none of the 11 variants tested in the conditional analyses completely removed the peak. However, two variants located in the *ABCC4* gene, at 70,053,503 and 70,052,385 bp, had significant effects on the 0/1 phenotype and ranked 8th and 9th, respectively, in the QTL peak. Thus, this gene is a strong candidate also for the 0/1 phenotype.

Conditional analyses of BTA13 included both the variant located at 63,502,566 bp (rs109570209), which had the most-significant effects on both the 0/1 and the 0/1/2 phenotypes, but also the neighboring variants located at 63,502,649 (rs380877320) and 63,500,701 (rs110002750), that had effects that were closest to the level of significance in Normande cows. None of these intergenic variants completely removed the signal for either of the two phenotypes.

Of the ten variants included in conditional analyses of BTA23, five completely removed the peak for both phenotypes. Three were located in genes, at 25,181,661 bp (rs209183236) in *ELOVL5* (intron), 26,733,104 bp (rs470365293) in ENSBTAG00000023541 (intron), and 28,085,410 bp (rs109539043) in *IER3* (upstream region). The two others were located in intergenic regions at 25,554,248 bp (rs210655104) and 28,012,299 bp (rs209284762).

## Discussion

Individual diagnostic tests for paratuberculosis are known to lack sensitivity and specificity, and these characteristics have been put forward as explanations for the low heritability of MAP-related traits that has been reported in the literature. Since the study of Settles et al. in 2009 [9] to the most recent publication in 2019, 13 GWAS analyses have been conducted for traits linked with bovine paratuberculosis [6–18]. These studies examined various numbers of animals and SNPs, and all succeeded in identifying genomic regions associated with MAP resistance/susceptibility. However, when taken together the results were generally poorly concordant. As mentioned before, many factors are likely contributing to these discrepancies, but one of the main ones, in particular, is the difficulty of properly characterizing the MAP status of cows due to (1) the long latency and incubation periods of the disease and (2) the lack of sensitivity of and concordance between milk ELISA and fecal culture.

In our study, we put a great deal of effort into defining accurate phenotypes. Control cows were chosen from affected herds and among those born in the same month as confirmed cases, this strategy being designed to increase the probability of their exposure to MAP. In addition, control cows were tested at least four times, as successive tests are known to be often inconsistent [5], and all cows had to be old enough for the latency period to be over, to limit the risk of false negative results. These constraints led to the exclusion of many animals from the study. Affected animals were selected from those that had previously been tested positive and their status was confirmed here with both ELISA and PCR tests; therefore, in order to include an affected cow for further analysis, we required both blood and fecal samples, and the lack of samples for many clinical cases resulted in their exclusion from the study. Because of all these conditions, only a small proportion (~ 5%) of all potential animals were included in the analysis. All confirmation tests were performed in a single laboratory with the same ELISA and PCR testing protocols throughout in order to make the results more reliable and comparable. Consequently, we are confident that the phenotypes used here accurately reflected the true status of the cows regarding MAP infection: not affected or affected with or without clinical signs.

The case–control design modified the distribution of the phenotypes and concentrated on the most extreme animals. In addition, it contributed to a much more balanced distribution of phenotypes than in the overall population of affected herds. Altogether, these choices resulted in heritability estimates that were much higher (around 50%) than those previously reported in the literature (3 to 27%) [6]. By removing the intermediate phenotypes, which were considered to be of uncertain status, our study likely overestimated heritability coefficients through the selection of individuals with extreme phenotypes. However, a part of our higher estimate can be explained by our focus on better-defined and thus likely more-heritable phenotypes. Nevertheless, these high estimates must be considered as specific to this selected study group, which strongly deviates from the general population, and are likely to be biased by this kind of selective genotyping.

All cows were genotyped with the Illumina SNP50 BeadChip. We intentionally did not use a low-density chip in order to reduce the potential impact of the reduced accuracy of imputation to 50 K. Imputation to HD, using 788 Holstein and 551 Normande key ancestors as a reference, is known to be very accurate [28]. We were then able to take advantage of the work of the 1000 Bull Genomes project to impute genotypes of the cows at the WGS level to directly identify candidate variants for resistance to paratuberculosis in Holstein and Normande cows. Our original design was intended to be the same in both Holstein and Normande breeds, with a full dataset for approximately 1500 cows in each breed. However, Holstein and Normande cows represent 64% and 9% of French herds, respectively, and it was much more difficult to recruit Normande than Holstein cows. For this reason, the final dataset was much smaller in the Normande (649) than in the Holstein (1644) breed, which clearly affected detection power; with a predefined significance threshold (−log_10_(*P*) ≥ 6), we identified more QTL regions with more significant effects in Holstein than in Normande cows. In the Holstein analysis, we detected 9 (0/1/2 phenotype) and 11 (0/1 phenotype) QTL, located on BTA12, 13, and 23, while only four significant QTL (0/1 phenotype), all located on BTA23, reached the significance level in the Normande analysis. Nevertheless, by analyzing both breeds independently, we were able to confirm the effects of the QTL located on BTA13 (with significant effects in Holstein and close to the significance threshold in Normande) and BTA23 (significant effects in both breeds). Another strategy would be to validate the three Holstein QTL in the Normande breed. Accordingly, the Bonferroni correction is limited to the number of candidate SNPs tested in the Normande breed. With this approach, the QTL on BTA13 would be validated (−log_10_(*P*) = 4.7) whereas the QTL on BTA12 would remain non-significant. However, additional data are needed in the Normande breed to increase the size of the population examined and improve the balance between numbers of case and control phenotypes.

By arbitrarily defining QTL regions in 1-Mbp-intervals, we identified several QTL on BTA12 and 23. However, for each of these chromosomes, conditional GWAS led to the identification of variants that each explained all the effects detected on BTA12 (rs41667085 in the *ABCC4* gene) and on BTA23 (rs209183236 in *ELOVL5*, rs4703655293 in ENSBTAG00000023541, rs109539043 in *IER3*, and rs210655104 and rs209284762 in intergenic regions). These results suggest that the identified variants could be the causative variants, or at least in strong LD with the causative variants. On both BTA12 and 23, we identified a large region that contains variants with significant effects. It could be due to the long-range LD that exists in these regions and/or, as shown by large segments with low imputation accuracy in QTL regions on BTA12 and 23, to local misassemblies of the bovine reference genome assembly UMD3.1. Moreover, for some QTL, conditional analyses failed to remove the peak. This could indicate that several causal mutations that affect resistance to paratuberculosis are located in this region.

Indeed, it is important to note that all these results were obtained using the UMD3.1 reference genome assembly, as for run6 of the 1000 Bull Genomes project, which was used for WGS imputation. When we compared the results obtained from different genome assemblies (ARS-UCD1.2 versus UMD3.1), the QTL regions on BTA13 and 23 were relatively well conserved, and we did not observe major changes for the variants located in these peaks. Instead, the QTL identified on BTA12 (a region of limited quality in UMD3.1) were located within a narrower peak in the ARS-UCD1.2 genome assembly (5 Mbp, versus 9.4 Mbp with UMD3.1 for the 0/1/2 phenotype), which was more consistent with the results that we obtained from the conditional analyses (a single QTL in the region).

Notably, although this study estimated relatively high heritability values for resistance to paratuberculosis, we found only a limited number of genomic regions associated with this trait in both breeds. Nevertheless, in Holstein cows, when we analyzed only the QTL with the most significant effects on each chromosome, the cumulative effects of the detected QTL explained 16 and 28% of the genetic variance of the 0/1 and 0/1/2 phenotypes, respectively. For both phenotypes, the QTL that explained the largest phenotypic variance was located on BTA13 and was responsible for 8% (0/1 phenotype) and 16% (0/1/2 phenotype) of the genetic variance, despite the fact that the resistance allele was present at a high frequency (0.91). Other QTL individually explained between 2 and 6% of the total phenotypic variance. Several previous GWAS analyses have reported QTL on BTA23 that are associated with resistance to MAP, in the vicinity of the MHC [7–9, 16, 18], while only one previous study, a meta-analysis conducted in US Holsteins based on 50 K SNP genotypes, found QTL at ~ 70 Mbp on BTA12 and ~ 65 Mbp on BTA13 [8]. McGovern et al. [18], who performed a GWAS on imputed whole-genome sequences, identified two SNPs associated with the humoral response to MAP on BTA13, at 62,037,755 bp and 66,373,805 bp, i.e. on either side of but quite far from the QTL we detected (63,497,960–63,506,532 bp) and outside its confidence interval. In this study, we did not find the QTL detected on the other chromosomes and, in particular, those located on BTA1, 6 or 7, which were the most commonly detected QTL for resistance to paratuberculosis in Holstein cows [6–12, 15, 16, 18, 36].

In each of the QTL regions detected here, we were able to identify genes of interest, some of which have been previously associated with traits related to the intestine or with responses to infection in mice, rats, or humans. Both *ABCC4* and *CBFA2T2* have been associated with intestinal inflammation and abnormal intestinal morphology (mucosa, goblet cell, enteroendocrine cell, or epithelium). Of all the genes located in the vicinity of the MHC on BTA23, *C4A*, ENSBTAG00000006864, and *IER3* have been linked to abnormal intestinal morphology or physiology, whereas *PKHD1*, ENSBTAG00000013919, ENSBTAG00000038397, and *SKIV2L* are involved in bowel diseases (Crohn’s disease, ulcerative colitis, syndromic diarrhea, gastrointestinal ulcer, or tricho-hepato-enteric-syndrome). Some of these genes have also been associated with an abnormal response to infection (*C4A*, ENSBTAG00000006864, and *IER3*), increased/decreased susceptibility to bacterial infection (*C4A* and ENSBTAG00000006864), or induced colitis (*ABCC4* and *IER3*). As bovine paratuberculosis is an enteric disease caused by the MAP bacterium leading to granulomatous enteritis, all of these genes are good functional candidates to explain inter-individual differences in resistance/susceptibility to paratuberculosis. In the QTL regions detected on BTA12, 13, and 23, the best candidates appear to be, respectively, *ABCC4*, *CBFA2T2*, and *IER3* because they contain (*ABCC4* and *IER3*) or are the closest (*CBFA2T2*) to the variants with the most significant effects.

In addition, we note here that our analyses of control/case (0/1) and control/non-clinical case/clinical case (0/1/2) phenotypes did not lead to exactly the same results. Although heritability estimates of both phenotypes were very similar in both breeds, there were differences in the GWAS results. In Holstein and Normande cows, depending on the phenotype, the number and identity of QTL detected differed (larger number for the 0/1 phenotype), as well as the functional candidate genes, probably reflecting different biological functions that contribute to each phenotype. For example, analysis of the 0/1 phenotype led to the detection of more QTL on BTA23 in both breeds and on BTA12 in Holsteins. In addition, on BTA12, QTL effects were more significant for the 0/1 phenotype than for the 0/1/2 phenotype. In contrast, the effects detected on BTA13 were much more significant for the 0/1/2 phenotype (−log_10_(*P*) = 38.1) than for the 0/1 phenotype (−log_10_(*P*) = 18.5). Since all the cows in this study were born in the same herd and within the same period as the infected cows, and therefore probably exposed to MAP, the control/case phenotype, which was mainly associated with the QTL located on BTA12 and 23, should reflect a cow’s ability to be resistant to MAP. Instead, distinguishing between non-clinical and clinical cases reflects the potential for a MAP-infected animal to postpone manifestation of clinical signs of the disease. Thus, these results appear to be concordant with the best functional candidate genes found in each QTL region, with the *ABCC4* (BTA12) and *IERC* (BTA23) genes appearing to be directly involved in responses to infection, whereas the *CBFA2T2* gene (BTA13) was previously found to be associated with intestinal inflammation or abnormal morphology in mice.

## Conclusions

In this study, we demonstrate that a focus on the most accurate phenotypes increases heritability estimates. These accurate phenotypes, combined with genotypes imputed to the whole-genome sequence level, made it possible to identify three chromosomal regions with important effects on resistance/susceptibility to MAP. In each of these regions, we were able to pinpoint one candidate gene that could be functionally related to MAP infection (*ABCC4*, *CBFA2T2*, and *IER3*) with candidate variants that could be either causal variants or in strong LD with the causal variants. Due to the large percentage of genetic variance explained, these QTL merit inclusion in future genomic evaluations with an appropriate weight. However, these QTL do not explain all of the relevant genetic variance, and the best model may very well end up containing QTL and markers from throughout the whole genome. Our results for the Holstein breed are very encouraging for strategies that aim at implementing selection for improved resistance to MAP. In the Normande breed, the study design was too limited to allow us to draw any general conclusions; efforts are underway to enlarge the sample pool in order to increase detection power.

## Supplementary information

**Additional file 1: Figure S1.** −log(*P*-value) plotted against the position of variants detected by GWAS (in grey) and conditional GWAS (GWAS_COJO; in blue).

## Data Availability

These data (genotypes and phenotypes) are part of a reference population used for genomic selection and have commercial value. Therefore, restrictions apply to the availability of these data, which are not publicly available. The authors can be contacted for a reasonable request.
