# Supplementary figures and images for: Identification of the ABCC4, IER3, and CBFA2T2 candidate genes for resistance to paratuberculosis from sequence-based GWAS in Holstein and Normande dairy cattle
Source: Genet Sel Evol. 2020 Mar 17;52:14. doi: 10.1186/s12711-020-00535-9 (PMC7077142; doi:10.1186/s12711-020-00535-9)

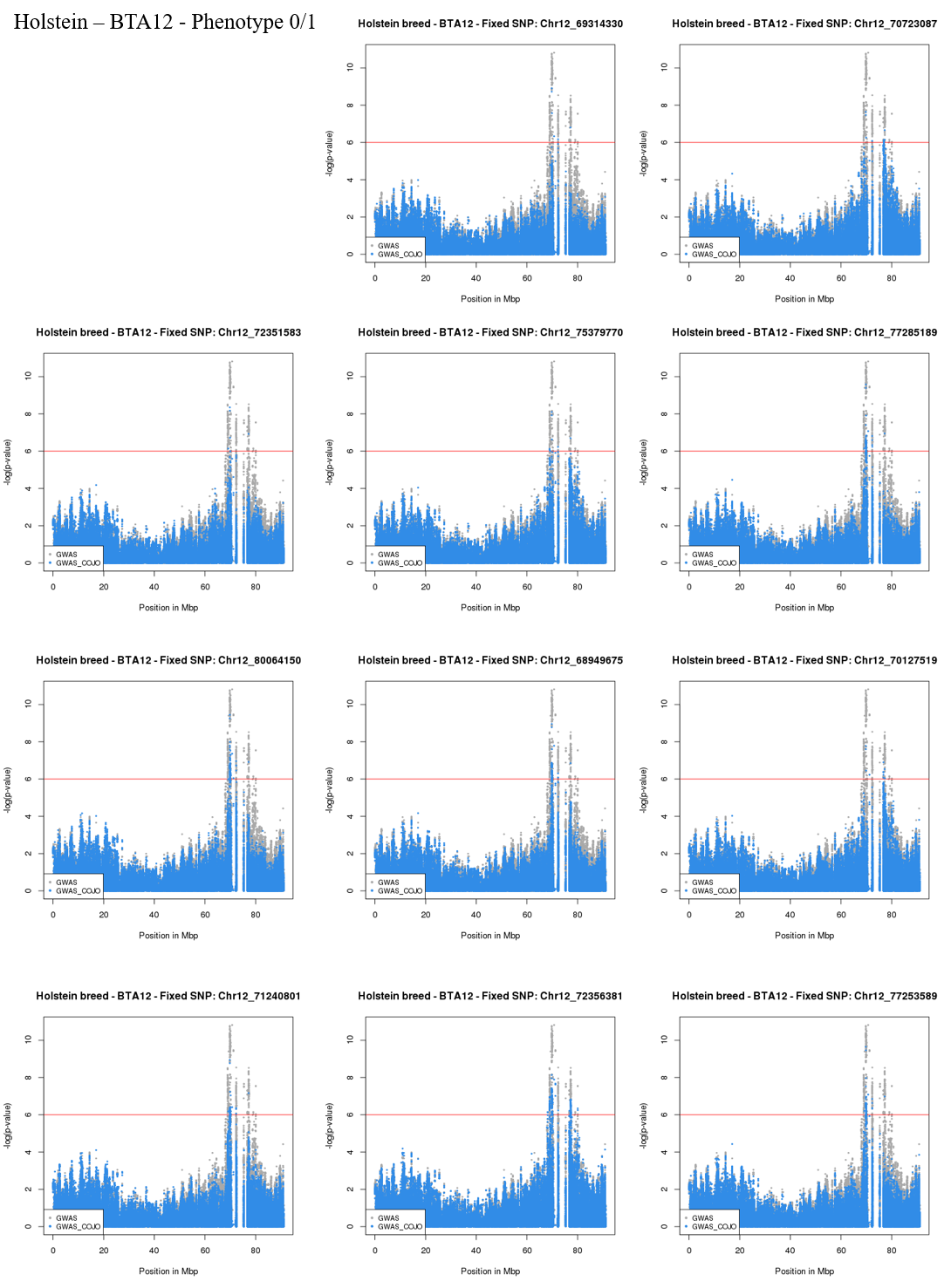

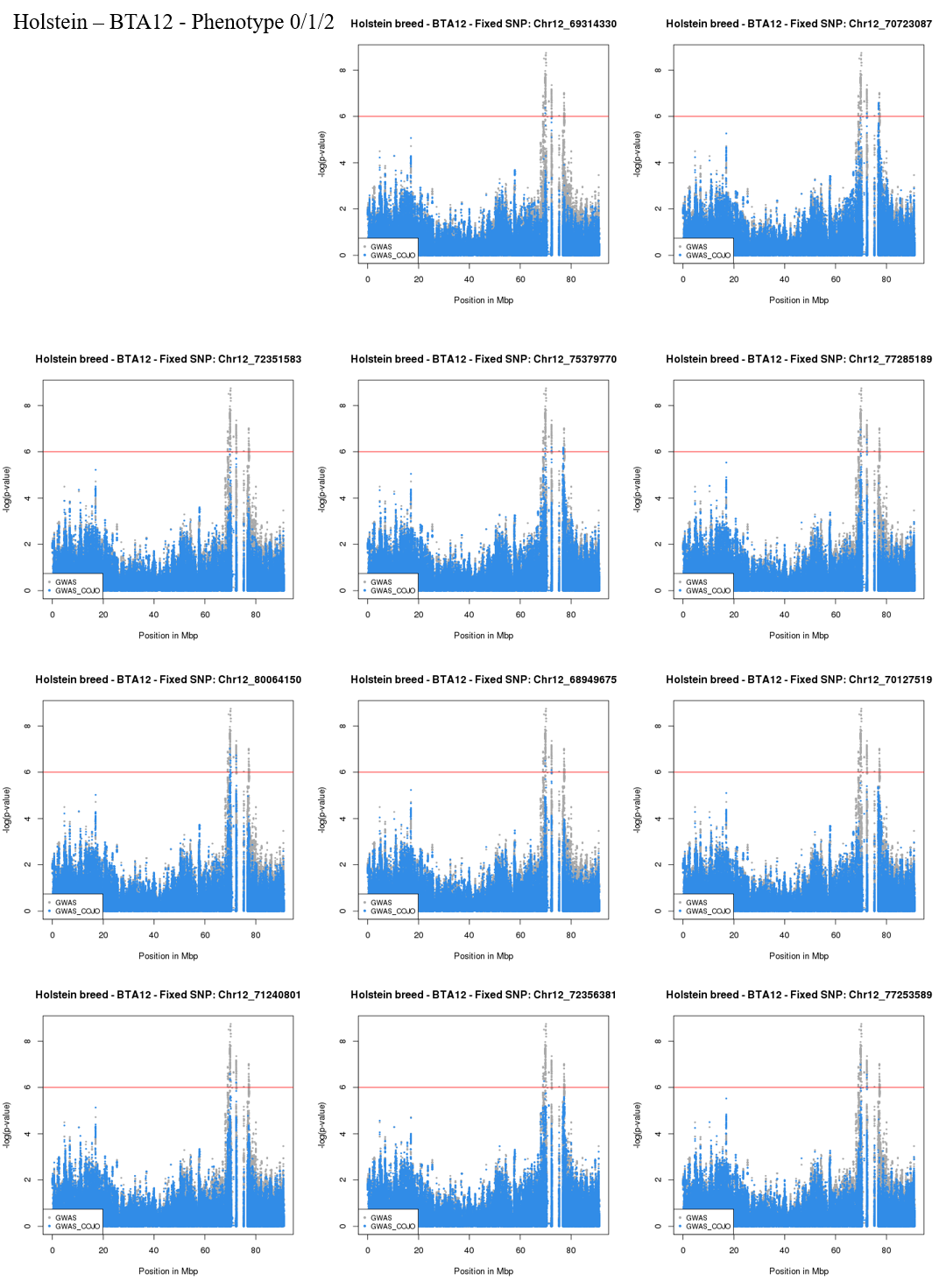

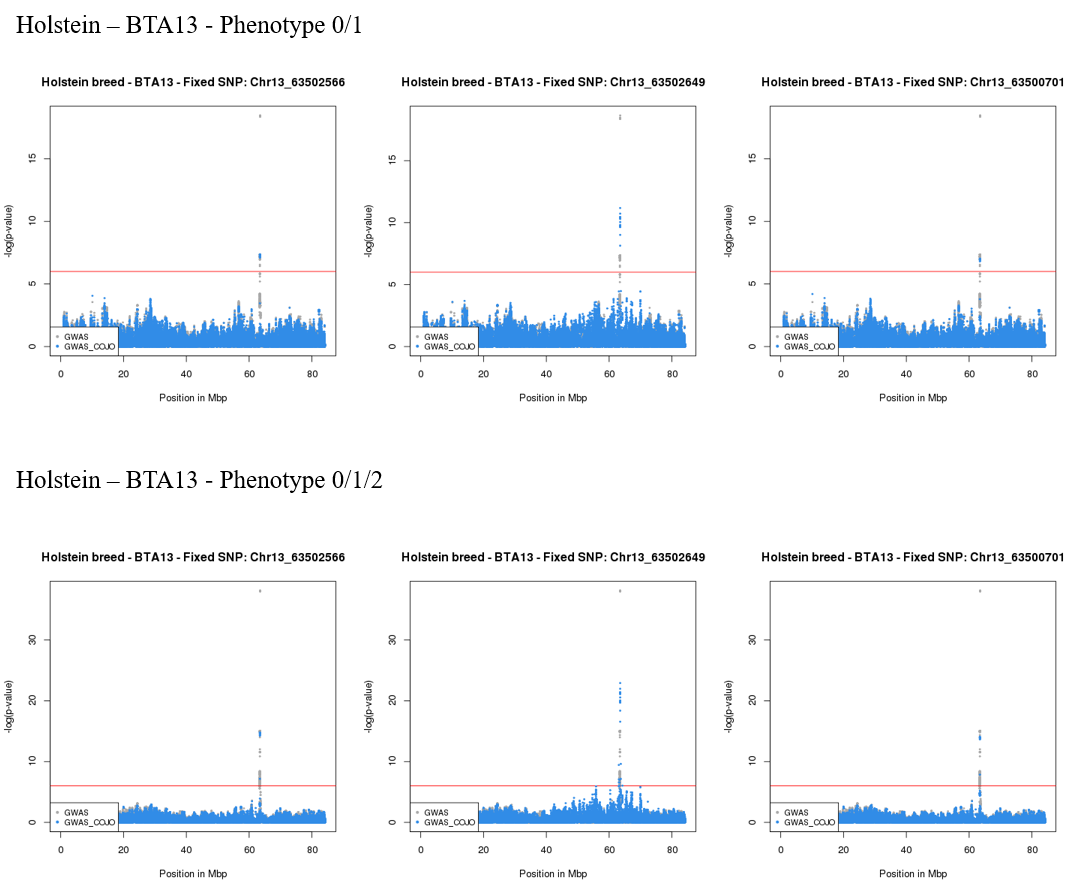

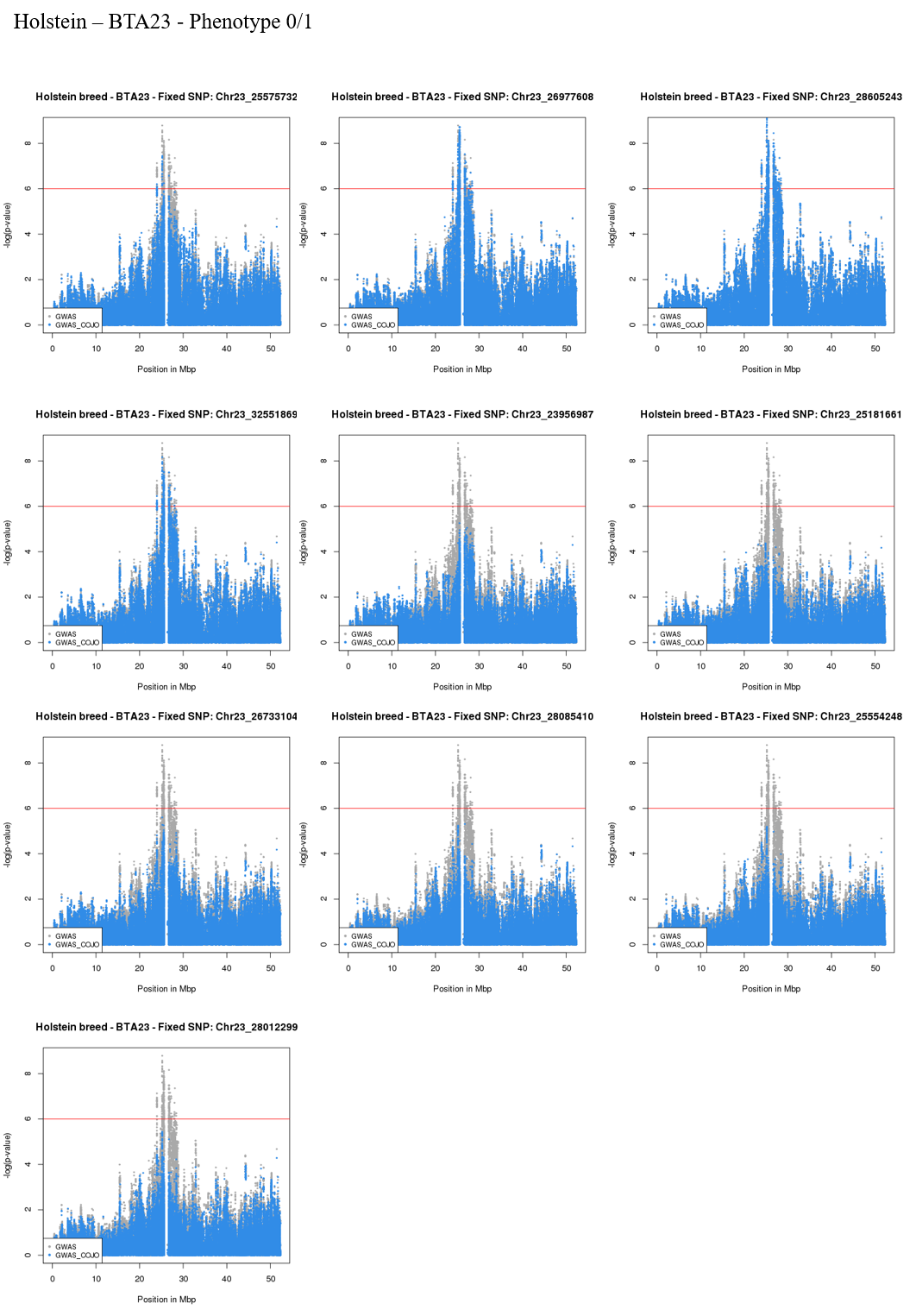

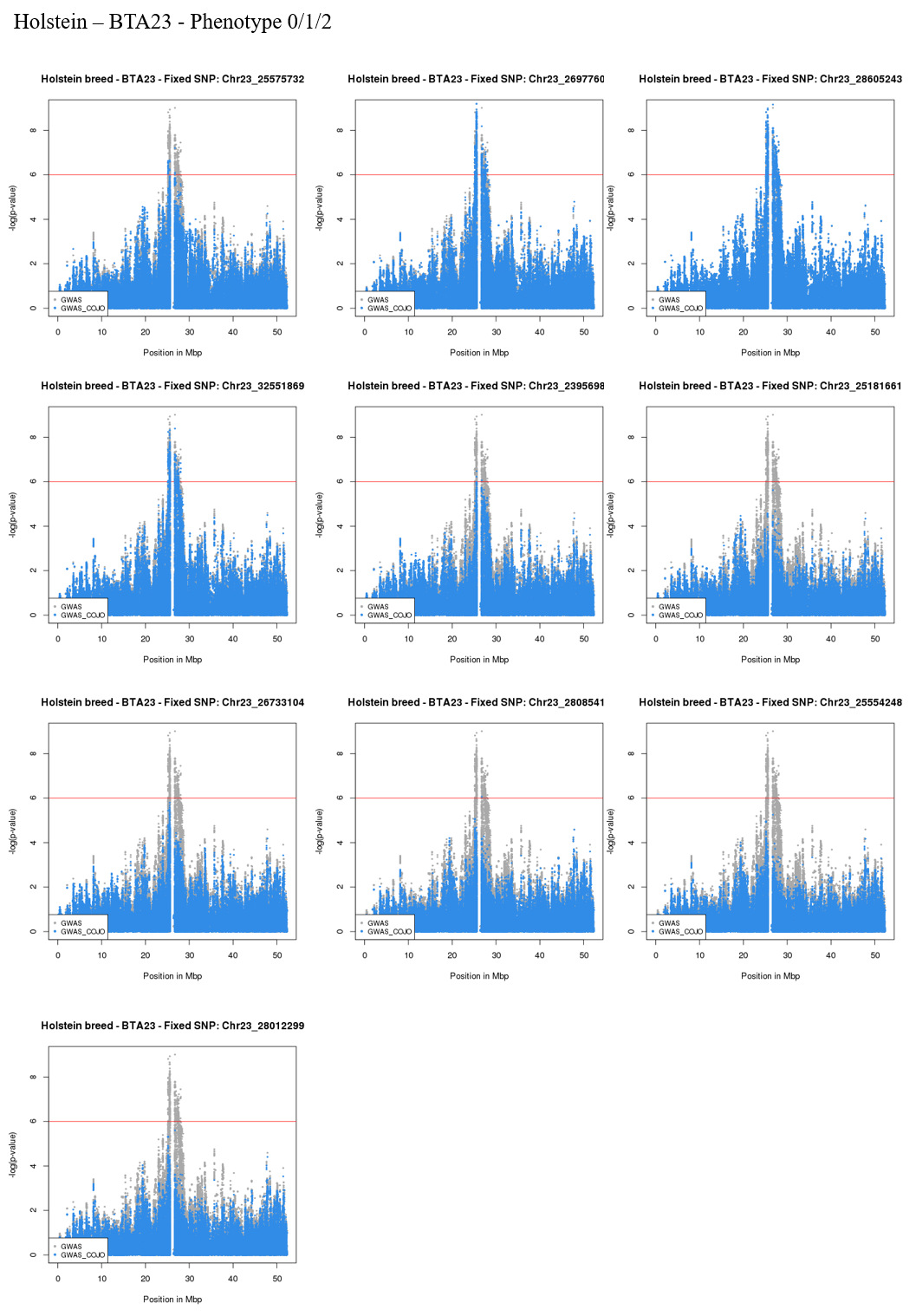

Supplement: Supplementary file 1 — Additional file 1: Figure S1. −log(P-value) plotted against the position of variants detected by GWAS (in grey) and conditional GWAS (GWAS_COJO; in blue). [file 12711_2020_535_MOESM1_ESM.docx]
